# Evaluation of Antioxidant Properties and Mineral Composition of Purslane (*Portulaca oleracea* L.) at Different Growth Stages

**DOI:** 10.3390/ijms130810257

**Published:** 2012-08-16

**Authors:** Md. Kamal Uddin, Abdul Shukor Juraimi, Md. Eaqub Ali, Mohd Razi Ismail

**Affiliations:** 1 Institute of Tropical Agriculture, University Putra Malaysia, Serdang 09800, Malaysia; E-Mail: razi@agri.upm.edu.my; 2 Department of Crop Science, Faculty of Agriculture, University Putra Malaysia, Serdang 09800, Malaysia; E-Mail: ashukor@agri.upm.edu.my; 3 Nanotechnology and Catalysis Research Center, University of Malaya, Kuala Lumpur 50603, Malaysia; E-Mail: eaqubali@gmail.com

**Keywords:** antioxidant, ferric-reducing antioxidant power (FRAP), ascorbic acid content (AAC), mineral composition, purslane

## Abstract

The main objective of this research was to appraise the changes in mineral content and antioxidant attributes of *Portulaca oleracea* over different growth stages. The antioxidant activity was measured using 1,1-diphenyl-2-picrylhydrazyl (DPPH), ferric-reducing antioxidant power (FRAP) assays. The iodine titration method was used to determine the ascorbic acid content (AAC). DPPH scavenging (IC_50_) capacity ranged from 1.30 ± 0.04 to 1.71 ± 0.04 mg/mL, while the ascorbic acid equivalent antioxidant activity (AEAC) values were 229.5 ± 7.9 to 319.3 ± 8.7 mg AA/100 g, total phenol content (TPC) varied from 174.5 ± 8.5 to 348.5 ± 7.9 mg GAE/100 g. AAC 60.5 ± 2.1 to 86.5 ± 3.9 mg/100 g and FRAP 1.8 ± 0.1 to 4.3 ± 0.1 mg GAE/g. There was good correlation between the results of TPC and AEAC, and between IC_50_ and FRAP assays (*r*^2^ > 0.9). The concentrations of Ca, Mg, K, Fe and Zn increased with plant maturity. Calcium (Ca) was negatively correlated with sodium (Na) and chloride (Cl), but positively correlated with magnesium (Mg), potassium (K), iron (Fe) and zinc (Zn). *Portulaca olerecea* cultivars could be used as a source of minerals and antioxidants, especially for functional food and nutraceutical applications.

## 1. Introduction

There are about 70 species of edible herbs in Malaysia which are locally known as “ulam” [1]. Some of these herbs are claimed to have high antioxidant properties as well as medicinal properties. The “weed” purslane (*Portulaca oleracea* L.) deserves special attention from agriculturalists and nutritionists alike. Purslane is a common weed in turfgrass areas as well as in field crops [2,3]. The mixture of phytochemicals present in many of these plants contributes to their protective and health effects [4].

Purslane has been studied in detail as a prolific weed, but very little is known about its production as a food crop and the effects of cultural conditions on its nutritional value, although there have been some studies carried out to determine the best cultural conditions to obtain higher levels of fatty acids [5] and lower levels of oxalic acid [6] in the leaves. Purslane (*Portulaca oleracea*), New Zealand spinach (*Tetragonia tetragonoides*) and cardoon (*Cynara cardunculus*) are promising crops for saline agriculture. The stems and leaves of the plant are succulent and edible with a slightly acidic and salty taste similar to spinach. In this regard, purslane is a reasonable choice due to its high nutritive and antioxidant properties as human food, animal feed and medical utilization. It is believed that the regular consumption of dietary antioxidants may reduce the risk of several serious diseases. Diets rich in fruits and vegetables have always been associated with health benefits, but their mechanism has become clear only in the recent decades [4].

Purslane comprises of a higher nutritive value than other vegetables due to its omega-3 fatty acid, α-tocopherol, ascorbic acid, β-carotene and glutathione rich shoots [7]. Recent research has shown that *P. oleracea* is a rich source of omega-3 fatty acids, which is important in preventing heart attack and strengthening the immune system [8]. The water extracts of *P. oleracea* showed no cytotoxic or genotoxic effects, and has been certified safe for daily consumption as a vegetable [9]. Such beneficial effects of this valuable weed might be ascribed to the presence of various bioactive and phenolic antioxidants. Consumption of flowers and vegetables high in antioxidants contribute to the prevention of degenerative processes caused by oxidative stress [10,11].

Although purslane has long been known in Malaysia, it is underutilized and considered a weed. To our knowledge, no data on nutritional quality of purslane have been published with regard to the plant maturity stages. Therefore, the objective of this study was to characterize the nutritional components and antioxidant activity of purslane at different maturity stages in an attempt to promote its use for human consumption.

## 2. Results and Discussion

### 2.1. Extraction Using Different Solvents

Results of the phenolic and flavonoid contents of purslane extracts obtained using different solvents are presented in Table 1. Water, 100% methanol and 50% ethanol extracts of the plant were examined to determine the best extraction solvent to be used in this study. Among the three solvents, the methanol extract yielded the highest TPC (360.3 mg GAE/100 g fresh weight). The TPC values of the 50% ethanol and water extracts were lower, being 276.3 and 142.8 mg GAE/100 g, respectively.

The flavonoid contents were also markedly higher in the methanolic extract with a value of 49.18 mg rutin equivalent/g DW compared to the ethanol extract at 41.3 mg rutin equivalent and water extract with a value of 28.7 mg rutin equivalent/g DW. Free radical scavenging activity of the methanolic extract was stronger than that of the boiling water extract, which was followed by ethanol. Phenolic compounds are widely distributed in plants [12] and have gained much attention because of their antioxidant activities and ability to scavenge free radicals.

Methanol is the most suitable solvent in the extraction of polyphenolic compounds from plant tissues, due to its ability to inhibit the action of polyphenol oxidase that causes the oxidation of polyphenols and its ease of evaporation compared to water [13]. Methanol extracts have been used in the study of *P. oleracea* flavonoids and some aspects of antioxidant activities [14,15]. On the contrary however, Cai *et al*. (2004) [16] reported that water extracts of powdered samples of *P. oleracea* at 80 °C for 20 min yielded higher total phenol content (0.6 g/100 g DW) than methanol extracts (0.4 g/100 g DW). This could be due to the hydrolysis of the glycosidic and ester bonds of the condensed flavonoids and hydrolysable tannins at near boiling temperature [17].

### 2.2. Growth of Purslane at Different Stages

Growth of shoots was recorded over 60 days by measuring fresh weight (FW), dry weight (DW) and shoot length at 15 day intervals (Table 2). The values of FW, DW and shoot length at the young stage (15 days) were lower than that of mature plants (60 days). The relative water content (RWC) of leaves was higher (90%) at 15 days but lower at 60 days (74%). The RWC decreased by 18% from the 15 day to 60 day old plants.

### 2.3. Comparative Study of Antioxidant at Different Plant Growth Stages

This study was conducted to determine the TPC content of the edible aerial parts of the plant at different growth stages (Table 3). The shoots were collected from 15, 30, 45 and 60 day old plants. The TPC value for the young shoots was significantly lower at 15 days than that of 30, 45 and 60 day old plants. The TPC at the mature growth stage of 60 days was slightly lower than in the plants at the developing stage. However, the AAC values did not show a significant decrease from the developing to the mature stage of the plant.

A higher level of TPC in the developing leaves could be due to the fact that protective compounds such as antioxidants are essential at the early growth stages. Plants at this stage are metabolically more active as they require a higher concentrations of essential compounds for growth. The lower TPC values in mature plants are attributed to oxidative stress as the plant is dying off [18]. Total phenol and flavonoid contents have been reported to be associated with antioxidant activity in various plants [19].

Components in leaf tissues can also change with maturity; for example, phenolic content decreases while contents of anthocyanins and other flavonoids increase. Young leaves in berry crops have higher contents of polyphenols compared to older leaves. Studies on the level of hesperidin at different stages of Citrus lemon growth showed increased levels in immature fruits which reached a maximum at the developing stage, and the level then decreased as the fruit grew to maturity [20,21]. It can be said therefore that the highest accumulation rate was at the young stages of development due to intense cellular division. The levels of polyphenolic compounds then decreased rapidly with age due to their dilution with leaf growth [22].

The values of TPC, AEAC and FRAP at 15 days were significantly lower than those at 30, 45 and 60 days (Table 3). The lowest values of TPC, AEAC and FRAP at 15 days were 174.5 ± 8.53 mg GAE/100 g, 229.5 ± 7.9 mg AA/100 g, and 1.8 ± 0.1 mg GAE/g, respectively. The highest values of TPC, AEAC and FRAP were found at 60 days and these values were 348.5 ± 7.9 mg GAE/100 g, 319.3± 8.7 mg AA/100 g, and 4.3 ± 0.1 mg GAE/g, respectively. The values of AAC and IC50 at 15 days were slightly lower than those at 30 and 45 days. The lowest values of AAC and IC50 were found at 60 days with values 60.5 ± 2.1 mg/100 g, and 1.30 ± 0.04 mg/mL, respectively. The highest values were found at 15 days; with values of 86.5 ± 7.9 mg/100 g, and 1.71 ± 0.04 mg/mL, respectively.

**Figure 1:**
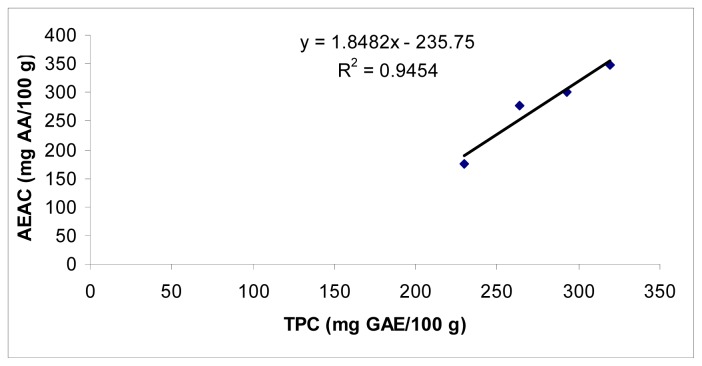
Correlation between total phenol content (TPC) and ascorbic acid equivalent antioxidant activity (AEAC) data in *Portulaca oleracea*.

**Figure 2:**
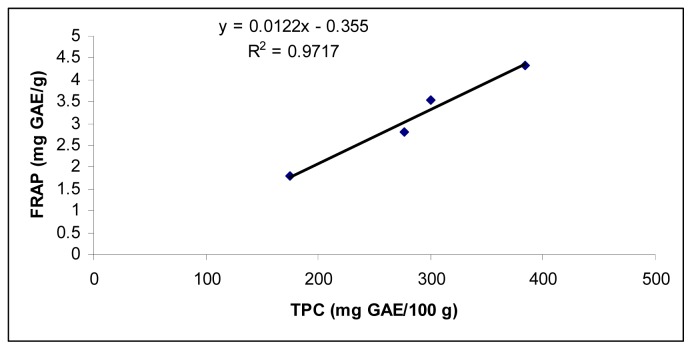
Correlation between TPC and ferric-reducing antioxidant power (FRAP) in *Portulaca oleracea*.

**Figure 3:**
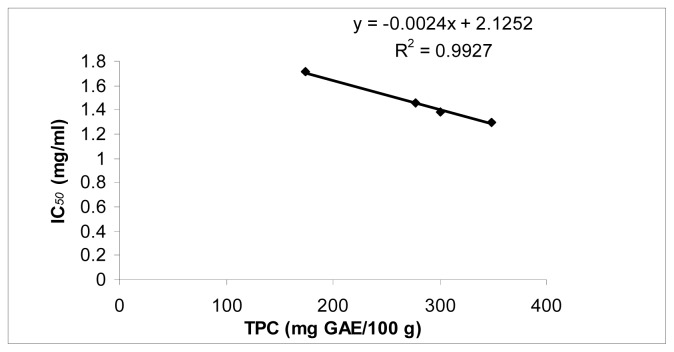
Correlation between TPC and IC_50_ in *Portulaca oleracea*.

**Figure 4:**
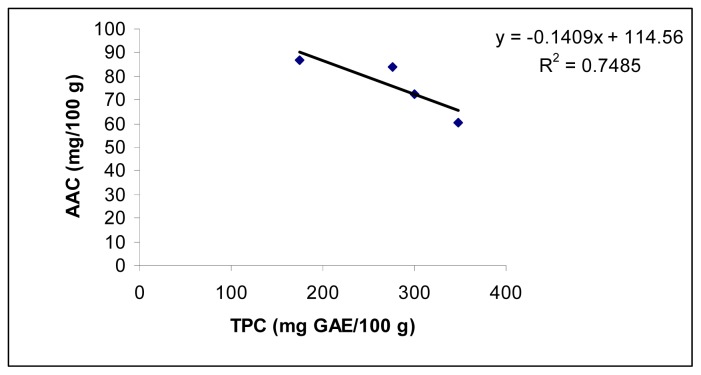
Correlation between TPC and ascorbic acid content (AAC) in *Portulaca oleracea*.

The IC_50_ ranged from 1.30 ± 0.04 to 1.71 ± 0.04 mg/mL, the AEAC values ranged from 229.5 ± 7.9 to 319.3 ± 8.7 mg AA/100 g, and 60.5 ± 2.1 to 86.5 ± 3.9 mg/100g and the FRAP values ranged from 1.8 ± 0.1 to 4.3 ± 0.1 mg GAE/g. The higher TPC value corresponded with higher AEAC and FRAPS values and lower IC_50_ values. For example, good correlation (*r*^2^ = 0.9454) between TPC of different cultivars and AEAC was observed (Figure 1). A similar relationship was also observed for FRAP which was highly correlated with TPC of purslane (*r*^2^ = 0.9717) (Figure 2). There was a negative linear relationship between IC50 and TPC (Figure 3). A similar relationship was found between AAC and TPC (Figure 4). The ascorbic acid content (ACC) also varies according to the different cultivars but does not correlate with the antioxidant activities. Different varieties, harvesting times and environmental conditions could also contribute to purslane composition [23].

### 2.4. Mineral Composition

Results depicted significant variations in Ca, Mg, Na, K, Fe, Zn and Cl in purslane leaves at different growth stages (Table 4). The values of Ca, Mg, K, Fe and Zn at the young stage (15 days) were lower than those of mature plants (60 days). The range of Ca, Mg, K, Fe and Zn from the young stage to mature plants was from 1612 ± 27 to 1945 ± 30 mmolkg^−1^ DW, 2127 ± 23 to 2443 ± 27 mmolkg^−1^ DW, 1257 ± 10 to 1526 ± 31 mmolkg^−1^ DW, 218 ± 8 to 262 ± 3 mmolkg^−1^ DW and 128 ± 2 to 160 ± 1 mmolkg^−1^ DW, respectively. On the other hand, the Na and Cl concentrations in leaves were higher at the young stage and lower at the mature stage. The Na and Cl concentrations ranged from 356 ± 4 to 278 ± 8 mmol kg^−1^ DW and 82 ± 2 to 53 ± 2 mmol kg^−1^ DW, respectively. Calcium (Ca) was negatively correlated with sodium (Na) and chloride (Cl), but positively correlated with magnesium (Mg), potassium (K), iron (Fe) and zinc (Zn) (Table 5). Iron was negatively correlated with Na and Cl, but positively correlated with Ca, Mg, K and Zn.

## 3. Experimental Section

### 3.1. Plant Materials

The experiment was conducted in a glasshouse at the Faculty of Agriculture, University Putra Malaysia. Young Purslane plants were collected from field and transplanted into 14 × 15 cm plastic pots filled with soil. Leaves of plants were collected for analysis of antioxidant properties every 15 days.

### 3.2. Preparation of Purslane Extracts

Leaf samples of *Portulaca oleracea* L. were extracted using three different solvents: ethanol, methanol and boiling water. Methanol and ethanol extracts were prepared by the method described in Crozier *et al*. (1997) [24]. Air-dried samples (0.5 g) were weighed and placed in 100 mL conical flasks, and 80% (v/v) ethanol or methanol (40 mL) was added, followed by addition of 6 M HCl (10 mL). The mixture was refluxed for 2 h at 90 °C and filtered through Whatman No. 1 filter paper (Whatman, UK), followed by evaporation of the filtrate under vacuum using a rotary evaporator (Buchi, Switzerland). The samples were stored at − 20 °C. The boiling water extraction was conducted using the method of Gulcin *et al.* (2004) [25].

### 3.3. Total Phenolic Content

The total phenolic compounds in the purslane extract was determined using the Folin-Ciocalteu reagent according to the method described by Halicia *et al.* (2005) [26]. Total phenolic content was expressed as milligrams of gallic acid equivalent (GAE) per gram dry weight (DW). Total phenol contents were determined as follows [27]. One and a half milliliters of Folin-Ciocalteu’s reagent (diluted 10 times) and 1.2 mL of Na_2_CO_3_ (7.5%, w/v) solution were added to 300 mL of plant extract. Mixtures were shaken and left to stand at room temperature for 30 min before measuring absorbance at 765 nm using a spectrophotometer (Anthelie Advanced 5 Secoman, France). The determination was done in triplicate. The total phenol content (TPC) was expressed as gallic acid equivalent in mg/100 g fresh plant material. Corrections were made for all TPC values in this study by subtracting the ascorbic acid content (AAC) from the total phenol value using the ascorbic acid standard curve.

### 3.4. Total Flavonoid Content

Total flavonoid compounds were determined using the aluminum chloride colorimetric assay based on Zhishen *et al.* (1999) [28]. Total flavonoid contents of the extracts were expressed as mg rutin equivalent/g dry weight (DW).

### 3.5. Antioxidant Activity (DPPH Free Radical Scavenging Activity)

The free radical scavenging activities of the extracts was determined as reported by Gulcin *et al*. (2004) [25]. The free radical scavenging activities of the tested samples was expressed as percentage of inhibition and calculated according to the following equation of Yen and Dudu, 1994. The results were expressed as % radical scavenging activity.

$$\%\;\text{radical scavenging activity}=(1-{\text{A}}_{\text{sample}}/{\text{A}}_{\text{control}})\times 100$$

IC_50_ which denotes the amount (mg) of plant in 1 mL solution required to reduce initial concentration of DPPH radicals by 50% was also calculated. Ascorbic acid was used as a standard and results were expressed as ascorbic acid equivalent antioxidant activity (AEAC) using the equation:

$$\text{AEAC }(\text{mg AA}/100\;\text{g})={\text{IC}}_{50}(\text{ascorbate})/{\text{IC}}_{50}(\text{sample})\times 100,000$$

### 3.6. Ferric Reducing Antioxidant Power (FRAP)

The ferric reducing property of the extracts was determined using the assay described by Yen and Chen (1994) [29]. The assay was carried out in triplicate. BHT and α-tocopherol were used as standard antioxidants.

One milliliter of extracts in different dilutions was added to 2.5 mL phosphate buffer (0.1 M, pH 6.6) and 2.5 mL potassium ferricyanide (1%, w/v). The mixture was then incubated in a water bath at 50 °C for 20 min followed by addition of 2.5 mL trichloroacetic acid (10%, w/v) solution. The contents of the tubes were mixed well and 2.5 mL of solution was removed from each tube. To this 2.5 mL solution, 2.5 mL water and 0.5 mL ferric chloride solution (0.1%, w/v) were added. The mixtures were allowed to stand for 30 min before absorbance measurements were taken at 700 nm. Triplicate tubes were prepared for each extract. The FRAP values expressed in mg GAE/g, were derived from the standard curve.

### 3.7. Chemical Analysis of Leaf Samples

Plant samples were dried in an oven at 70 °C for 72 h. Oven-dried samples of Purslane were ground and stored in plastic vials. The K, Na, Ca and Mg contents were analyzed using the digestion method (Ma and Zua, 1984) [30] and determined using an Atomic Absorption Spectrophotometer (AAS; Perkin Elmer, 5100, USA).

### 3.8. Statistical Analysis

Data were analyzed using the analysis of variance procedure in SAS (version 17). Significance among means were determined using the LSD test at *p* = 0.05 level.

## 4. Conclusions

Mature plants of *Portulaca olerecea* had higher TPC and antioxidant activities than plants at the immature stages. In particular, 60-day old plants had very high TPC and antioxidant activities as assessed by the 2,2-diphenyl-1-picrylhydrazyl (DPPH) test and the ferric-reducing antioxidant power (FRAP) assay. The values of Ca, Mg, K, Fe and Zn at the mature stage were higher than young plants. The findings of this study are important for selecting *Portulaca olerecea* cultivars at the appropriate maturity stage for use as a source of valuable minerals and antioxidants, especially for functional food and nutraceutical applications.

## Figures and Tables

**Table 1:** Total phenolic and flavonoid content of *Portulaca oleracea*.

Solvent	Total phenolic content 1	Total flavonoid content 2
Ethanol	276.8 ± 5.5	41.30 ± 4.0
Methanol	360.3 ± 8.9	49.2 ± 3.4
Water	142.8 ± 8.7	28.7 ± 2.1

1 mg GAE/100 g DW, mg rutin equivalent g^−1^ DW.

2 mg rutin equivalent g^−1^ DW.

**Table 2:** Fresh weight, dry weight, shoot length and relative water content of *Portulaca oleracea* at different growth stages.

Day intervals	FW (g)	DW (g)	Shoot length (cm)	RWC (%)
15	11.97 ± 1.10	0.93 ± 0.05	4.12 ± 0.42	90 ± 2.69
30	20.52 ± 0.83	1.23 ± 0.037	8.50 ± 0.64	88 ± 2.53
45	26.30 ± 1.06	1.33 ± 0.023	14.75 ± 0.85	79 ± 2.41
60	29.15 ± 0.73	1.97 ± 0.053	20.25 ± 0.86	74 ± 2.71

**Table 3:** Total phenols, ascorbic acid content, DPPH free radical scavenging activity (IC_50_ value), ascorbic acid content (AAC), ascorbic acid equivalent antioxidant activity (AEAC) and ferric-reducing antioxidant power (FRAP) data for *Portulaca oleracea* at the different growth stages.

Day intervals	TPC (mg GAE/100 g)	AAC (mg/100 g)	IC_50_ (mg/mL)	AEAC (mg AA/100 g)	FRAP (mg GAE/g)
15	174.5 ± 8.5	86.5 ± 3.9	1.71 ± 0.04	229.5 ± 7.9	1.8 ± 0.1
30	276.8 ± 5.5	84.0 ± 4.2	1.46 ± 0.03	263.8 ± 8.3	2.8 ± 0.1
45	300.5 ± 6.2	72.3 ± 2.7	1.38 ± 0.01	293.0 ± 8.2	3.6 ± 0.1
60	348.5 ± 7.9	60.5 ± 2.1	1.30 ± 0.04	319.3 ± 8.7	4.3 ± 0.1

**Table 4:** Mineral concentrations in *Portulaca oleracea* at different growth stages (mmol kg^−1^ DW).

Minerals	15 Day	30 Day	45 Day	60 Day
Ca	1612 ± 27	1742 ± 22	1892 ± 27	1945 ± 30
Mg	2127 ± 23	2196 ± 16	2250 ± 21	2443 ± 27
Na	356 ± 4	332 ± 7	306 ± 3	278 ± 8
K	1257 ± 10	1289 ± 7	1323 ± 11	1526 ± 31
Fe	218 ± 8	248 ± 5	252 ± 4	262 ± 3
Zn	128 ± 2	136 ± 1	142 ± 2	160 ± 1
Cl	82 ± 2	71 ± 1	62 ± 2	53 ± 2

**Table 5:** Pearson Correlation matrix for mineral composition.

Factor	Ca	Mg	Na	K	Cl	Fe	Zn
Ca	1						
Mg	0.799 **	1					
Na	−0.872 **	−0.896 **	1				
K	0.735 **	0.892 **	−0.837 **	1			
Cl	−0.901 **	−0.829 **	0.892 **	−0.755 **	1		
Fe	0.836 **	0.619 **	−0.759 **	0.683 **	−0.720 **	1	
Zn	0.813 **	0.924 **	−0.826 **	0.888 **	−0.865 **	0.690 **	1

** Correlations significant at the 0.01 level.
